# “We are never taught anything about the elderly.” Establishing the gap in elderly health care competencies in nursing education in Uganda

**DOI:** 10.1186/s12912-022-00936-9

**Published:** 2022-06-21

**Authors:** Faith Nawagi, John Mukisa, Josephine Nambi Najjuma, Rose C. Nabirye

**Affiliations:** 1 Makerere University College of Health Sciences (MaKCHS), School of Medicine, Kampala, Uganda; 2 Makerere University College of Health Sciences (MaKCHS), School of Biomedical Sciences, Kampala, Uganda; 3 Mbarara University of Science and Technology (MUST), Faculty of Medicine, Kampala, Uganda; 4 Busitema University, Faculty of Health Sciences, Kampala, Uganda

**Keywords:** Elderly health care, Training, Uganda, Nursing, Competencies

## Abstract

**Background:**

Nurses contribute the largest portion of Uganda’s health workforce providing care to individuals of all ages and communities. However, despite the growing number of the elderly population in Uganda with improved life expectancy, there is hardly any study that has looked at the elderly health care competencies in the nursing training programs at various levels. This paper provides an overview of the gaps in elderly health care competencies in nursing education in Uganda.

**Methods:**

We conducted a descriptive qualitative cross-sectional study that involved document review, Key Informant Interviews (KIIs) with nursing leaders, and Focus Group Discussions ( FGDs) with faculty at all levels of nursing training and nurses in practice. Data was analyzed using latent and manifest content analysis with Open Code software 4.03. Common categories were identified and incorporated into a matrix to create themes.

**Results:**

Almost all the curricula and minimum standards for training nurses at certificate, diploma, and degree levels lack a module and nursing competencies on elderly nursing care. This is aggravated by a lack of faculty trained in elderly health care skills, and a lack of specialized wards for nursing elderly care clinical training among others.

**Conclusions:**

There is hardly any elderly health care training module and elderly nursing competencies at all levels of nursing training in Uganda.

## Introduction

Aging remains a growing demographic characteristic of the twenty-first century given the improved health care systems and survival globally [1]. The 2019 global population aging report highlights that there are currently 962 million older adults (≥ 60 years), and this is expected to double by 2050 [2]. One in 11 people is over the age of 65 years and this is expected to be 1 in every 6 people by 2050, with the number of those aged 80 and over to triple in the next 30 years [2]. Furthermore, over 80% of this population will be living in developing countries with over 11% living in Africa [3]. This is further evidenced by the increasing life expectancy in many African countries with the majority being ≥ 60 years [4].

Although there is no standardized cut-off to define old age, the United Nations (UN), African Union (AU), and the Ministry of Gender, Labor, and Social Development (MoGLSD) in Uganda use age 60 and above to refer to older persons [5]. In Uganda, the number of older persons has increased from 1.1million in 2002 to 1.5million in 2020 and is expected to increase to 6 million by 2050 [5]. Furthermore, life expectancy in Uganda has risen to 63 years over the past ten years [6].

Older persons in Uganda constitute the poorest members of society. About 64% of them survive on less than $1USD a day [7]. They lack access to regular income, and the majority do not benefit from the national social security provisions since it’s available to those in formal employment, yet the majority of the Ugandans are self-employed. Most live in inadequate housing with others being homeless, especially in Kampala, where a few are street beggars and sleeping on the roadsides at the night, with only one meal, which leaves them emaciated and exposed to diseases [8].

Furthermore, the elderly suffer from many diseases ranging from joint pains, Chronic Non-Communicable Diseases (NCDs) to recurrent malaria [9]. These are commonly addressed in the public hospitals in the rural areas that face lots of stock-outs which in the end makes care costly and unaffordable [10]. Many suffer from multiple forms of disability. Physical disability accounts for 56% of the cases, while visual impairment accounts for 39% [11]. These leave the elderly dependent on others due to the inability to engage in income-generating and self-sustaining activities [12].

Despite the challenges faced by the elderly, there is scanty scientific literature documented about elderly health competencies in the training of health professional students of which nursing is part. With the current Sustainable Development Goal (SDG) 3 that encourages health for all through universal health coverage, the elderly health care sector, especially in Africa, remains understudied and with very few interventions. In Uganda, Africa, and globally, nurses contribute to the largest health workforce, especially in elderly health care [13]. The current available elderly care competencies and assessments in the various levels of nursing have been implemented in developed countries. A study done in Uganda by Ssensamba et al. found a low readiness for public health facilities to provide elderly health care [14]. This was attributed to the low availability of geriatric health care leadership and departments in the hospitals, low financing, low human resources with geriatric health care skills, and lack of health management information systems to monitor elderly health care needs [14]. Lack of any health care skill of which geriatrics is part is largely a factor of training in the various health qualification training at different levels. It is this gap in human resources with elderly health care skills from Ssensambas's study that prompted our study aim inline to nursing training programs given nurses contribute to the largest health workforce at the frontline in Uganda and Africa at large. Notably, there is barely any study that has looked into the preparedness of the nursing training to equip nursing students at various levels of training with elderly health care competencies in Uganda and Africa at large. This qualitative cross-sectional study, therefore, aimed to address this knowledge gap by establishing the gaps in elderly health care competencies in nursing education in Uganda.

## Methodology

### Study design

This was a descriptive cross-sectional study using qualitative methodology. This involved document review, Key Informant Interviews (KIIs) with leaders of the nursing profession, and Focused Group Discussions (FGDs) with faculty at certificate, diploma, and bachelors’ level and in-service nurses. All methods were performed in accordance with the relevant guidelines and regulations in Uganda.

### Study setting

The curriculum followed by certificate and Diploma level was obtained from the Uganda Nurses and Midwives Examination (UNMEB) board website [15] Nursing training at the certificate and diploma level in Uganda is supervised by the Ministry of Education and Sports through the sector of Business, Technical Vocational Education and Training (BTVET) [16]. There are currently 107 nursing schools licensed to train nurses at certificate and diploma levels in Uganda [15]. These use the same curriculum and sit the same exams administered by the Uganda Nurses and Midwives Board [15]. While data collection for certificate and diploma levels was from one source since there is a homogeneity of curricula. that of undergraduate nursing training was collected from independent universities since different universities tend to have a slight difference in their Bachelor of Nursing curriculum. The bachelor's level of nursing training in Uganda occurs in universities and is overseen by the National Council of Higher Education (NCHE) [17]. This has set minimum standards for Bachelors of Science in Nursing which are adopted by all the 15 universities training nurses at the undergraduate level [18]. The minimum standards for the undergraduate bachelor's nursing training from the NCHE) were also obtained online and reviewed. Out of the 15 universities that train nursing at the undergraduate level, only 6 where included to represent the various public or private setting in addition to the various regions in Uganda given the limitations in funding. These include Makerere University, Agakhan University, Mbarara University of Science and Technology (MUST), Kampala International University (KIU), Lira University (LU), and Clarke International University (CIU). However, we did not get a response from Makerere and Agakhan University. The various universities are in Kampala, Mbarara, Ishaka, and Lira districts respectively. MUST and LU are government-funded universities whereas KIU and CIU are private universities. The institutions are accredited to train nursing at an undergraduate level by the NCHE. All the undergraduate programs at these universities have been existent for more than 5 years. The in-service nurses were obtained from central, North, East, and western Uganda from various public or private health care facilities and, at different levels from health center 2 to regional referral hospital level.

### Study population

The study population for document review included curricula, and minimum standards for nursing training at certificate, diploma, and degree levels. Faculty training nurses at various levels of nursing training i.e., certificate, diploma, and degree level, and in-service nurses from Central, East, North, and Western Uganda were included. KIs included current and recently outgoing nursing leaders that have served in the profession for several years and in various roles from the Uganda Nurses and Midwives Examination Board (UNMEB), Uganda Nurses and Midwives Council (UNMC), Federation of Uganda Nurses and Midwives, Ministry of Health and Nursing Now Uganda.

### Sampling method and recruitment of participants

Purposive sampling was used in this study to include nurse educators, nurse leaders, and in-service nurses. This was used given the nature of the study being qualitative which required us to gather responses from the best fit participants to get better in-depth insights on the subject.

### Study tools

All tools were administered in English and no translation was required since the study is targeting the literate community and English is used as the official language of instruction for the nursing profession in Uganda. Below is the description of the various study tools used.

### Nursing Training Competency Assessment Tool for Elderly Health Care

The data collection checklist was adopted and developed using the Gerontological Nursing Competencies and Standards of Practice 2010 by the Canada Gerontological Nursing Association (CGNA). This was used simply because there are no tools that have been developed for this purpose in Uganda and Africa at large.

### FGD Guide

This was adapted from the need assessment conducted for the Council of Ontario Universities on the perceptions of practitioners and practitioner organizations about gaps and required competencies for seniors’ care among health and social care graduates and workers. This was validated and pretested for the study setting before use.

### Data collection

Data collection was carried out between Dec 21- Feb 2022. The Curriculum review data collection was done online. Certificate and Diploma level Curricula and the minimum standards for bachelor’s training, are available as public documents on the BTVET, UNMEB, and NCHE websites [15] [16] [17]. These were downloaded and assessed for elderly nursing competencies using the checklist developed in reference to the CGNA. For the various universities, 1-h zoom meetings were set up with each of the heads of departments of nursing to jointly review the curriculum guided by the checklist developed by the CGNA. Participants were approached via telephone calls, thereafter, consent forms and interview guides were emailed to the participants at least 2 days before the interview. For nurse educators, FGD participants were grouped depending on the level of the students they are teaching.

Seven FGDs were carried out with nursing faculty training at certificate, Diploma, and bachelor's levels in central (1), East (2), North (2), and Western (2) Uganda. Furthermore, 4 FGDs were carried out with nurses in practice in central (1), east (1), north (1), and western (1) Uganda from public and private health care settings at different levels from health centers 2 to regional referral levels. These nurses were serving in internal medicine, emergency, surgery, and outpatient departments where elderly patients are interfaced with. We also carried out 5 key Informant Interviews with current and recently outgoing nursing leaders that have served in the profession for several years and in various roles from the Uganda Nurses and Midwives Examination Board, Uganda Nurses and Midwives Council, Federation of Uganda Nurses and Midwives, Ministry of health and Nursing Now. A minimum of 5 and a maximum of 8 participants were included in each FGD to enable effective participation. Participant responses from the FGDs and KIs were audio-recorded and later transcribed verbatim for effective analysis. Each of the participants was sent the consent form and FGD/KI guide before the meeting to enable them to get acquainted with the questions and do more substantive preparations. The FGDs/KIIs were conducted by FN, RCN, JNN, and trained qualitative researcher assistants. The research assistants had a nursing background, had prior experience in conducting FGDs/KIIs, and they were both male and female. The researchers were not known to most of the participants. The meetings were audial recorded using the zoom application to enable effective text analysis. The researcher posed the questions and invited responses from participants and once each participant responded, the next question was asked till the completion of the questions. The interviewers took field notes. The time required to do the FGDs was 1.5 h. While the point of saturation determined the number of participants in this study, efforts to ensure equal representation from all regions in Uganda were made. The FGDs / KIIs in this study were conducted virtually online using zoom. This was in consideration of the COVID-19 situation but also much more cost-effective to collect the data from the various parts of the country.

### Data analysis

We used Open Code software 4.03[19] to perform the qualitative data analysis. A trained social anthropologist and the principal investigator (who has had training in qualitative methodology) carried out the final data analysis. The data was analyzed using both latent and manifest content analysis. The latent analysis involved interpretation of the underlying meanings of the text which requires further abstraction and is more in-depth, while manifest involved analysis of visible components of the text [20]. The research team and the data analysis team read the transcripts several times to identify meaning units from the transcripts. The units were then condensed and coded. The codes were further categorized from which emerging sub-themes and themes were generated. Qualitative data summaries and quotes from the study participants were presented. No transcripts were sent to the FGD participants after the closure of data collection. Document review of the different curricula of the nurses at the different levels of diploma, certificate, and degree obtained from various institutions in Uganda was done. The findings from the review were presented as narratives and proportion tables using frequencies and percentages.

## Results

This section describes the findings obtained from a review of the various curriculum and minimum standards for nursing training at various levels in Uganda. It also describes the results obtained from the various FGDS and KIIs with nurses in practice, faculty for a certificate, diploma, and degree level, coupled with leaders of the various nursing bodies in Uganda respectively.

### Nursing training curricula and minimum standards for geriatric nursing competencies and modules

Table 1 shows the various curriculum reviewed for the various levels.

**Table 1:** Various Curriculum and Minimum Standards Guiding Nursing Training at Various levels in Uganda *N* = 7

Curriculum Reviewed	Level of Training Applied	Presence of Geriatric Nursing Competencies /Module
Curriculum for certificate nursing 2018	Certificate	Absent
Curriculum and examination syllabus for Diploma Nursing training in Uganda 2018	Diploma	Absent
Bachelor of Science in Nursing Minimum standards 2019	Bachelors	Absent
Lira University	Bachelors	Absent
Mbarara University of Science and Technology	Bachelors	Present
Clarke International University Nursing	Bachelors	Absent
Kampala International University	Absent	Absent

Both the national curriculum for certificate and diploma nursing did not have a module on geriatric health care. Of the 4 undergraduate, nursing curricula reviewed only one (MUST) had a module and competencies in geriatric nursing. The curricula and minimum standards in **Table ****1** were assessed for any competencies in geriatric nursing using a checklist developed using the Canada Gerontological Nursing Association (CGNA) geriatric nursing competencies.

As shown in Table 2, most of the checklist geriatric nursing competencies had higher percentages in the “No” category. Furthermore, the variable competencies “ Intervene to assist older adults and their support network to achieve personal goals, based on the analysis of the living environment and availability of community resources” and “Recognize the complex interaction of acute and chronic co-morbid physical and mental conditions and associated treatments common to older adults” had 100% of the documents in the “NO” or absent category.

**Table 2:** Findings from Curriculum Review for Elderly Nursing competencies using the CGNA checklist. *N* = 7

Geriatric nursing competencies of the CGNA to be acquired by nursing students	No, N (%)	Yes, N (%)
Incorporate professional attitudes, values, and expectations about physical and mental aging in the provision of patient-centered care for older adults and their families	6 (85.7)	1 (14.3)
Assess barriers for older adults in receiving, understanding, and giving information	6 (85.7)	1 (14.3)
Use valid and reliable assessment tools to guide nursing practice for older adults	6 (85.7)	1 (14.3)
Assess the living environment as it relates to the functional, physical, cognitive, psychological, and social needs of older adults	6 (85.7)	1 (14.3)
Intervene to assist older adults and their support network to achieve personal goals, based on the analysis of the living environment and availability of community resources	7 (100.0)	0 (0.0)
Identify actual or potential mistreatment (physical, mental, or financial abuse, and/or self-neglect) in older adults and refer appropriately	6 (85.7)	1 (14.3)
Implement strategies and use online guidelines to prevent and/or identify and manage geriatric syndromes	7 (100.0)	0 (0.0)
Recognize and respect the variations of care, the increased complexity, and the increased use of healthcare resources inherent in caring for older adults	6 (85.7)	1 (14.3)
Recognize the complex interaction of acute and chronic co-morbid physical and mental conditions and associated treatments common to older adults	7 (100.0)	0 (0.0)
Compare models of care that promote safe, quality physical and mental health care for older adults such as PACE, NICHE, Guided Care, Culture Change, and Transitional Care Models	6 (85.7)	1 (14.3)
Facilitate ethical, non-coercive decision-making by older adults and/or families/caregivers for maintaining everyday living, receiving treatment, initiating advance directives, and implementing end-of-life care	6 (85.7)	1 (14.3)
Promote adherence to the evidence-based practice of providing restraint-free care (both physical and chemical restraints	6 (85.7)	1 (14.3)
Integrate leadership and communication techniques that foster discussion and reflection on the extent to which diversity (among nurses, nurse assistive personnel, therapists, physicians, and patients) has the potential to impact the care of older adults	6 (85.7)	1 (14.3)
Facilitate safe and effective transitions across levels of care, including acute, community-based, and long-term care (e.g., home, assisted living, hospice, nursing homes) for older adults and their families	6 (85.7)	1 (14.3)
Plan patient-centered care with consideration for mental and physical health and well-being of informal and formal caregivers of older adults	6 (85.7)	1 (14.3)
Advocate for timely and appropriate palliative and hospice care for older adults with physical and cognitive impairments	6 (85.7)	1 (14.3)

### Findings from FGDS and KIIs on elderly nursing training competencies in Uganda

#### Characteristics of participants in the FGDs and KIIs

Table 3 represents the characteristics of the participants in FGDs and KIIs. The median age of the participants was 33 years and more than half of the participants 57(61.3%) were female.

**Table 3:** Social demographic characteristics of the FGDs and KIIs participants *N* = 93

Characteristic	Frequency (N)	Percentage(%)	Summary statistic
FGD participants			
**Age**			
Median, Interquartile range			33.0, 30–38
**Sex**			
Female	57	61.3	
Male	36	38.7	
**Region**			
Central	15	16.1	
East	30	32.3	
North	26	28.0	
West	22	23.6	
**Level of training**			
Bachelors	25	26.9	
Masters	24	25.8	
Diploma	35	37.6	
Certificate	9	9.7	
**Years of experience**			
Median, Interquartile range			8.0, 6–11
Minimum	1		
Maximum	40		

**Fig. 1:**
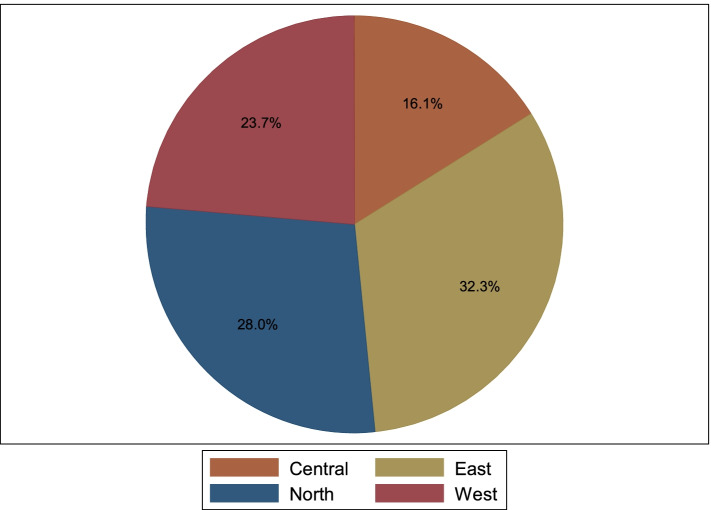
Pie chart showing the distribution of study participants by region

The distribution of participants by regions in Uganda is shown in Fig. 1 below.

The results are grouped based on the Gaps/barriers to the elderly care training and possible models of delivering geriatric nursing competencies to nurses.

### Barriers to elderly care training identified in FGDs and KIIs

The gaps in elderly nursing care training were identified in almost all the curriculum, nursing training period, and post-school professional period. Six themes were generated to extensively describe the gaps and various quotes were cited as shown below to establish the in-depth description.

#### Theme 1: Elderly care nursing training is missing in guiding documents

##### Elderly care is not part of the nursing curriculum at all levels

Most of the study participants in the FGDs and KIIs mentioned that the curriculums in the different nursing institutions at all levels or cadres of nursing training were generally lacking competencies in the management of the elderly individuals in Ugandan communities. Nurses also emphasized that there were no avenues known to them where they could receive elderly education if they wished to acquire it.

*“No, I have not seen any emphasis on elderly care or heard it in any of the curricula from years back up to now. The elderly have not been looked at with the critical eye”-FGD I-nurse Central Uganda*

*“Most of the competencies are missing in our curriculum, 100%” FGD-Nurse Educators Central Uganda*

*“There are no courses available in Uganda to train the students on geriatric nursing care”- FGD1-nurse – -Western Uganda*

*“When we look at the training of nurses, there is not enough emphasis on geriatric nursing. Even in the curriculum, there is nowhere in the curriculum where the elderly are catered for. It makes the individuals feel that being old is a bad thing contrary to our Bible teaching.”.FGD1- Nurses- Eastern Uganda.*

Even among the teachers, there were gaps in elderly care in their training curriculum later translated to less information on elderly care passed on to their students. This was noted as a lack of knowledge for elderly care.

*“As nurses, we lack knowledge on how to care for the elderly because it is not part of our training curriculum” FGD-4-nurses -Northern Uganda*

*“Based on my experience as a post-BSN (Bachelor of Nursing) trainee, we do not have anywhere in our training curriculum where we had a focus on elderly care practices. I do not remember any course unit, course work, or assessment. FGD-1-Nurses-Central Uganda*

*Even when I look at the certificate and diploma training curriculum and in-servicing training, the competencies are at least not favoring learning elderly care practice.”- KI, nurse leader, Central Uganda*

*“We did not train the nurses (while in school) to handle the elderly people. We have little knowledge about the elderly conditions, which leads to mismanagement and gaps in care” –FGD-2-diploma and certificate teacher Western Uganda*

Participants also mentioned that the inadequacies in training were not only limited to the theoretical aspects of the nursing training but extended to the practical sessions in the wards. In addition, the respondents highlighted gaps in training exposure for the nurses post-training, to continue offering care for elderly patients.

*“I am involved in training general diploma nurses and midwives; I can categorically say that there are no specific areas where we teach our students about elderly care. We also assign our students to the general wards and not assign them any elderly specific wards due to their absence”- KI Nurse leader Central Uganda*

##### Lack of clinical guidelines on elderly health care

There are no guidelines to inform clinical care of the elderly in our Ugandan setting. Nurses practice care for the elderly according to the care for adults. The participants mentioned;

*“We do not have any national guidelines to use to train the nurses about elderly care. There is no policy to guide geriatric care in Uganda, which makes the provisional of care to the elderly a bit messed up” FGD 3–Nurse-Educator Northern Uganda*

*“The nursing care in Uganda is still developing. There are no policies to inform the geriatric nursing care from the certificate, diploma and degree level of training”-FGD-3-Nurse Educator, Western Uganda.*

#### Theme 2: Poor staff attitudes and communication skills

##### Negative attitude towards the elderly

It was mentioned that attitude determines how well a nurse serves the individual before them. An unfavorable attitude was stated as a gap in the provision of care to the elderly.

*“The nurses at the moment are harsh towards the elders and do not want to give them care”-FGD-3-Nurses-s-Eastern- Uganda*

*“Our elderly are not taken care of since we have bad attitudes towards them. We think that we are too smart to support them. We fear to talk to them about their health because we are not sure whether they will follow the instructions due to the age differences”- FGD-1-Nurse Educators-Western Uganda*

In some situations, there are gaps in teaching the nursing students about the required attitude to provide care to the geriatric patients.

*“In our settings, the elderly have particular needs and the nurses need to understand them. We have not yet been keen to inculcate the right attitude to look after the elderly with their nitty-gritty like mood changes”. FGD-1-Nurse Educators -Western Uganda*

##### Lack of proper communication skills in the elderly care setting

The inadequacy of communication skills of nurses toward elderly patients was highlighted as a challenge to the provision of care for the elderly.

*“There is a lack of skills in communicating with the elderly. You find the nurses on the ward, shouting at the elderly people, talking very badly to them sometimes yet they could speak to them better” FGD-1-Nurse educator Eastern Uganda*

#### Theme 3: Inadequate infrastructure for the provision of elderly care

##### Lack of infrastructure to provide specialized elderly care

The lack of dedicated wards for elderly patients has led to challenges for the nurses to provide care for the elderly. They are unable to tailor care to the elderly patients on these wards since there are many numbers of elderly. Three participants mentioned:

*“There is no specific ward for the elderly people. At the moment, they mix with the rest of the adults which makes the provision of social support, and mental health care to the elderly hard”- FGD-3-Nurse-Educator-northern Uganda*

*“In the hospitals where the patients are treated, we do not see any specific areas where the elderly are kept. This is mainly because of high patient volumes. There is no specific care for the elderly”- FGD-1-Nurse-Educator-Central Uganda*

*“In the private and government hospitals, we do not have anything (rooms) regarding the availability of care for the elderly”-FGD-2-Nurses-Educators-Eastern Uganda.*

In the hospital, setting, there are wards for pediatrics and no wards for the elderly, which makes them lack privacy to say their issues and have specific care. In some instances, this led to a lack of privacy for the elderly in most government facilities in Uganda due to the absence of dedicated wards for the elderly.

*“The elderly tend to mind so much about confidentiality. As long as they realize that someone one young is the caretaker/helper, they remain reserved and not open up about their condition”-FGD-4-Nurses-Western Uganda*

#### Theme 4: Limited numbers of trainers with training in elderly nursing care

##### Limited numbers of trainers with training in elderly nursing care

The respondents mentioned that the limited nursing schools’ staff trained in elderly care was a key gap in the existence of elderly care courses in the curriculum.

*“We have one or two staff that have adequate knowledge to implement the geriatric nursing curriculum at our institution. They are also very busy. So, we are stuck with the implementation of the program”- FGD-2-nurse-Educators- Western Uganda*

“*There are few trained nurses on the ward to take care of the elderly. The senior nurses do not know what to do with the elderly and leave the work to the junior nurses. Sometimes you go to the ward and there are 2 nurses on duty for 30 patients, which makes the nurses overloaded, strained, and frustrated. Even we (nurse trainers), we are deficient in some elderly care practices.” FGD-2-Nurse Educator-Central Uganda*

#### Theme 5: Possible models of delivering competencies in elderly nursing

We identified pre-service (before leaving nursing training institutions) and in-service (after leaving nursing training institutions) ways of delivering competencies in geriatric care as described below:

#### For pre-service training

##### Incorporate and integrate elderly nursing into the curriculum

To most of the participants, the incorporation of elderly nursing course units in the curriculum through all levels of the nurse's training was considered a good model of enriching future nurses with knowledge on the subject.

*“There is an opportunity to integrate elderly care in nursing training at each of these levels. Since the enrolled and diploma nurses make up most of the cadres, we could design a course unit of maybe 3 CU credit units. This will bridge the knowledge gap”- FGD-1-Nurse-Educator Central Uganda*

Other participants suggested that there would be designated wards in the different hospitals to facilitate bedside teaching for learning and skills transfer regarding elderly nursing care.

*“I think there needs to be “special corners” (wards) for the elderly in the hospitals to enable the nurses to broaden the scope of learning about the care for the elderly-FGD-1-Nurse Educators-Eastern Uganda.*

##### Offer training to nursing trainers

Moreover, the teachers and trainers would also need to be trained in elderly nursing care and therefore stimulate the transfer of knowledge to nursing trainees. One respondent said:

*“We need to train the mentors (preceptors) onwards and tutors about elderly nursing so that they can pass on the information to the nursing trainees.”- FGD-2-Nurse-Educator Central Uganda*

*“We need to train the tutors about elderly nursing care since they do not know what to teach the nurses”-FGD2-nurse-Educator-Eastern Uganda*

##### Designing community practicum at the elder’s home

Participants in the KIIs and FGDs echoed the need for hands-on training in geriatric care nursing for all nursing cadres. They proposed that it would be best if this were conducted in the nursing homes or newly created geriatric wards at their training hospitals to facilitate learning and knowledge transfer. One participant noted.

*“We have two-course units like community nursing and nursing the child. We can also encourage the teachers to send the students to elders in their respective communities to enable their reflective learning on the elderly care”- FGD-2-Nurse Educator Northern Uganda*

#### For in-service training:

Most participants expressed the idea that already qualified nurses should be targeted for routine training sessions and short courses on elderly nursing care to improve their competencies. These sessions would be helpful and impactful for elderly care in the communities.

*“ We can design CPD (Continuous professional development) and routine training modules that are accredited by the Nursing council that would be required before renewal of licenses.” FGD-1-Nurse educator in Central Uganda*

Other participants suggested that short courses for elderly nursing could be designed for the nurses to learn about elderly care in their areas.

“ *I think we can design short courses like those of three months or two weeks so that they can learn about elderly care.”-FGD-2-nurse Educator-Eastern Uganda*

## Discussion

The current increment in life expectancy in sub-Saharan Africa is attributed to improvement in health care systems, economic development, globalization, reduction in infant mortality, and political stability among others in the region in the past 2 decades [21, 22]. In Uganda, the population of older persons (>60years) contributes 4.3% of the population [5]. Despite being the minority group, 17% of all households in Uganda have an older person [5]. The growth is expected to double from 1.5m to 6.2million in the next 3 decades [5]. Despite these projections in Uganda, significant gaps in supporting the social and health care needs of the elderly in Uganda and Africa at large are existent. The Ugandan Government is making efforts to address the needs of the elderly through the Social Assistance Grant for Empowerment (SAGE) program that provides financial social protection to the elderly under the Ministry of Gender Labor and Social Development [23]. However, the amount (8USD) given is too low compared to the cost of living in Uganda.

We found out that there was a gap in nurses' training in elderly health care competencies at all levels in Uganda. This has also been reported in other studies in Africa. A study done by Dotchin et al. showed a significant lack of geriatric training at undergraduate and specialist levels across all health disciplines in sub-Saharan Africa [24]. As regards health care for the elderly, a study done in Uganda by Ssensamba et al. found a low readiness for public health facilities to provide elderly health care [14]. This was attributed to the low availability of geriatric health care leadership and departments in the hospitals, low financing, low human resources with geriatric health care skills, and lack of health management information systems to monitor elderly health care needs [14].

Nurses contribute the biggest percentage of the health workforce in Uganda and Africa at large [25]. These are required to be adequately trained to provide health care to all Ugandan population categories including the elderly. In this study, we reviewed the curriculum and minimum standards used to guide nursing training at undergraduate, diploma, and certificate levels to establish the existence of geriatric nursing competencies and independent modules. In this study, we found a lack of an independent module in geriatric nursing and geriatric nursing competencies in both the certificate and diploma nursing curriculum currently being followed for training. Furthermore, the FGDs and KIIs with faculty at different levels, nurses in practice, and leadership showed inadequate clinical training infrastructure and limited numbers of skilled trainers to conduct elderly health care training. This has led to inadequate nursing elderly health care in the Ugandan health care system due to insufficient knowledge and skills.

These findings are similar to those found by Abudu-Birresborn et al. where the nursing students and the nurses at the frontline had insufficient knowledge of the care of the elderly largely due to a lack of elderly health care training modules and competencies in the nursing curriculum of low and middle-income countries [26]. Furthermore, from this study the lack of elderly health care skills among the practicing nurses was attributed to elderly care not being part of the nursing curriculum at all levels, Lack of clinical guidelines in elderly care, Lack of integrated community geriatric care practice, Negative attitudes by nurses towards the elderly, Lack of proper communication skills in the elderly care setting, Lack of infrastructure to provide geriatric care, and Limited numbers of trainers with training in geriatric nursing care. These findings relate to the finding by Ssensamba et al. where they found low human resources skilled in elderly care, and low infrastructure availability to provide elderly health care in Uganda [14]. On the contrary, a scoping review on preparing nurses and nursing students to care for older adults in lower and middle-income countries showed a positive attitude among the nurses towards the care of the elderly [26]. However, in by Abudu-Birresborn et al. study, it is important to note that the positive attitude was attributed to being knowledgeable about geriatric care, unlike in this study where the nurses were not knowledgeable about geriatric nursing care [26].

We found that the minimum standards for Bachelor of Science in nursing currently being followed lack geriatric nursing care competencies and an independent elderly health care module. However, despite the existence of the minimum standards, each university administers its own exams, and the curricula slightly defer from one university to another. This is the reason why we went ahead to review the curricula followed by various university training institutions that could be accessed. Almost all of the curricula lacked an independent module and geriatric health care competencies except that followed by Mbarara University of Science and Technology at the undergraduate level. These findings are similar to those found by Naidoo et al. where there was a lack of a module and emphasis on geriatric health care competencies in the nursing undergraduate curriculum at the University of KwaZulu Natal in South Africa [27].

Although this study found gaps in elderly health care, it is important to note that the elderly in Uganda are treated as any other adult seeking care and thus attending care in the various hospital systems and departments in place. Most elderly suffer from chronic illnesses like diabetes and hypertension among others and access care through the respective hospital departments addressing these illnesses [1]. However, the lack of skilled health care providers in elderly care, elderly clinical guidelines, and hospital departments dedicated to the elderly leads to inadequate health care for the elderly with gaps in geriatric assessment, diagnosis, and treatment priorities [8]. It is also important to note that access to health care by the elderly in Uganda is determined by household income status, NCDS, mobility limitations, and severity of illness [10]. This, therefore, means that there is a significant number of elderly that fail to access health care and thus are being cared for by their families.

In Ugandan culture, the elderly play a key role in the propagation of cultural norms and are meant to live and be cared for by their families [28]. This is evidenced by findings from Tam et al. where the family and close relatives of the elderly play a significant role in providing care for the elderly in the form of meeting their basic daily needs, hygiene, nutrition, and caring for them when with a chronic illness like strokes and other non-communicable diseases [8]. This, therefore, means that as we aim to address the gap in elderly health care training in nursing education in Uganda, consideration should be done to include the community/families through an integrated community elderly health care training approach to enable nurses to skill the various families with elderly care skills but also build a relationship with them to enable them to develop the confidence to send their elderly to hospitals when the need arises.

Nevertheless, despite the significant gaps in nursing elderly health care training, enabling factors such as the existence of nursing training schools, and nurses willing to be skilled to train and provide elderly health care are available. These factors can be used as a starting point to develop nursing elderly health care competencies as other enablers needed like clinical training centers and clinical guidelines among others are being addressed.

## Conclusion

There is hardly any elderly health care training module and elderly nursing competencies in all levels of nursing training in Uganda.

### Recommendations

There is a need to develop a curriculum for elderly health nursing care to train nurses in training at all levels (Certificate, Diploma, and bachelor) and in-service nurses in Uganda.

### Quality Control

The trustworthiness and rigor of this study given its qualitative nature were observed. For credibility Prolonged engagement of the participants, having the research team review the findings, and double data analysis was done. Furthermore, the collection of data from different participants and institutions was done to ensure triangulation. A detailed description of the qualitative data collection and analysis process was done to ensure transferability in similar contexts elsewhere. To observe the dependability of the findings a statistical software was used to derive findings. To observe confirmability the study findings were reviewed by the study team for accuracy and alignment with the study objectives.

### Limitations

This study was qualitative and thus prone to participant acquiescence bias. However, the research team ensured open-ended questions and provided enough time to provide in-depth responses with an emphasis correct understanding of the questions by the participants.

## Data Availability

'The tools and data set for this study are available upon reasonable request from the corresponding author.
